# Silencing of *Synuclein-γ* inhibits human cervical cancer through the AKT signaling pathway

**DOI:** 10.1186/s11658-019-0172-y

**Published:** 2019-07-10

**Authors:** Chunnian Zhang, Liqin Gu, Xiafang Li, Jianzhong Wang

**Affiliations:** Department of Gynaecology, Ganzhou People’s Hospital of Jiangxi Province, No. 18, Meiguan Avenue, Ganzhou city, 341000 Jiangxi Province China

**Keywords:** *Synuclein-γ*, Knockdown, Cervical cancer, Growth, AKT

## Abstract

**Background:**

*Synuclein-γ* has been demonstrated to be highly expressed in various human cancers including cervical cancer, and has been shown to play a critical role in tumor aggressiveness. We aimed to investigate the role of *Synuclein-γ* in human cervical cancer in vitro and in vivo.

**Method:**

Reverse transcription-quantitative polymerase chain reaction assay and Western blot assay were used to detect the mRNA and protein expression, respectively. 3-(4,5-dimethylthiazol-2-yl)-2,5-diphenyltetrazolium bromide assay and *colony formation assay* were performed to measure the viabilities of cancer cells. Flow cytometry assay was used to detect the cell cycle and apoptosis. Moreover, an animal experiment was performed to evaluate the biological behavior of *Synuclein-γ* in vivo.

**Results:**

In the current study, we found that *Synuclein-γ* was obviously over-expressed in cervical cancer tissues compared to the adjacent non-cancer tissues. Cervical cancer cells transfected with *Synuclein-γ* siRNA demonstrated significant inhibition of cancer proliferation (*P* < 0.01), cell cycle arrest at G0/G1 phase, and cell apoptosis (*P* < 0.05). Moreover, down-regulation of *Synuclein-γ* significantly inhibited cervical cancer growth in vivo. In addition, protein levels of *AKT*, c-Myc and Cyclin D1 were much lower in the *Synuclein-γ* siRNA-treated groups than that in the control group.

**Conclusions:**

*Synuclein-γ* inhibition reduced cervical cancer tumor growth through the AKT pathway. This effect represented a therapeutic opportunity and provided a novel target for cervical cancer treatment.

## Background

As one of the most common malignant tumors among women, cervical cancer has a tendency to affect young persons, with an estimated 470,000 new cases and 200,000 deaths each year in the world [1–3]. The usual treatments for cervical cancer are surgery and radiotherapy, and the overall 5-year survival rate is approximately 40% [4]. While metastasis or recurrence usually happens in advanced patients, the prognosis remains poor [5]. Thus, novel effective therapeutic strategies are urgently needed and further exploration of the underlying mechanism is urgently required.

*Synuclein-γ* (*SNCG*) is the third member of the synuclein family [6], which is strongly associated with malignant progression and distant metastasis in different types of cancer. *SNCG* protein is abnormally expressed in a high percentage in various malignant tumor tissues including liver, breast, ovarian, prostate and colon cancer, while it is rarely expressed in tumor-matched non-neoplastic adjacent tissues [7, 8]. Cumulative findings suggest that *SNCG* might be a potential biomarker in cancer progression.

In this study, we transfected cancer cells with a small interfering (si)RNA targeting the *SNCG* gene, and that efficiently inhibited *SNCG* expression at the messenger (m)RNA and protein expression level in cervical cancer cell lines. We firstly demonstrated that the inhibition of *SNCG* results in reduction of cell viability, cell apoptosis, and cell cycle being arrested at G0/G1 phase through the Akt signaling pathway. Furthermore, knockdown of *SNCG* inhibited tumor growth of cervical cancer in vivo. In conclusion, *SNCG* might suppress tumor growth, thus being a potential therapeutic target for cervical cancer.

## Methods

### Cervical cancer clinical samples and cell culture

This study was approved by the Research and Ethics Review Committee of Ganzhou People’s Hospital of Jiangxi Province following the Declaration of Helsinki principles. Written informed consent was obtained from every subject. Thirty cervical cancer samples and corresponding adjacent normal tissues were harvested from female patients in Ganzhou People’s Hospital of Jiangxi Province between April 2014 and April 2015, in which diagnosis were confirmed by pathologists. All specimens were collected before patients received any treatment such as chemotherapy, radiotherapy and surgery. All samples were immediately frozen in tubes after removal and stored at − 80 °C. Histological classifications and clinical staging were based on the classification system by the International Federation of Gynecology and Obstetrics (International Federation of Gynecology and Obstetrics Cancer Committee; FIGO, 2009) (29). The clinical characteristics of all patients are shown in Table 1. The high or low expression levels of SNCG were defined by the median level of expression.

**Table 1:** Relationships between SNCG expression and clinicopathological characteristics in cervical cancer patients

Characteristics	SNCG expression (30)		*P*
	Low (n)	High (n)	
Age			
< 50	6	7	0.532
≥ 50	6	11	
Tumor size (cm)			
≤ 4	10	9	0.021*
> 4	3	8	
FIGO staging			
I-II	9	8	0.016*
III–IV	3	10	
Histological grade			
Well differentiated	5	6	0.577
Moderately to Poorly differentiated	7	12	
Pelvic lymph node metastasis			
No	11	10	0.021*
Yes	2	7	

Human cervical cancer cell line (HeLa, SiHa) was obtained from the Chinese Academy of Sciences. Human cervical epithelial cells (HCerEpiC) were obtained from Shanghai Institute of Cell Biology (Shanghai, China) and maintained under standard conditions. All cells were grown in DMEM (Gibco, CA, USA) supplemented with 10% fetal bovine serum (FBS; Excell Bio, Shanghai, China), 100 units/mL penicillin G and 100 μg/mL streptomycin (Gibco) in a water-saturated atmosphere of 5% CO_2_ at 37 °C. The medium was changed every 2–3 days.

### Depletion of SNCG by siRNA

Small interfering RNA (siRNA) vectors targeting the human *SNCG* gene and a control vector carrying a sequence unrelated to the human gene were obtained from GeneChem Co., Ltd. (Shanghai, China). *SNCG* or control siRNA was transfected into cancer cells using Lipofectamine 2000 (Invitrogen), according to the manufacturer’s instructions. A total of 3 experimental groups were designed as follows: the *SNCG* siRNA vector-transfected cells (*SNCG* siRNA group), negative control vector-transfected cells (NC group) and untransfected cells (CON group).

### Reverse transcription-quantitative polymerase chain reaction (RT-qPCR)

Total RNA was extracted using TRIzol reagent (Invitrogen) according to the manufacturer’s protocols. Total RNA (1 μg) was reverse-transcribed using SuperScript II reverse transcriptase (Invitrogen) in a total volume of 20 μL. The reaction mixtures were incubated at 37 °C for 60 min, 95 °C for 5 min and then held at 4 °C. For the PCR reaction, a mixture containing 25 ng cDNA, 7.5 μM primer each (GeneChem Co.), 12.5 μL PCR Master (Invitrogen), and nuclease-free water in a total volume of 25 μL was prepared. *β-actin* mRNA levels were quantified to normalize expression levels. RT-qPCR was performed using the SYBR-Green PCR Core Reagents kit (Thermo, MA, USA) as follows: 1 cycle of denaturation at 94 °C for 2 min, followed by 35 cycles at 94 °C for 0.5 min, 60 °C for 0.5 min and 72 °C for 0.5 min, and a final extension step at 72 °C for 10 min. Real-time detection of SYBR Green fluorescence was conducted using an ABI StepOnePlus Real-Time PCR System (Thermo Fisher Scientific, MA, USA).

The specific primer pairs were as follows: *SNCG*, forward primer, 5′-ATGGATGTCTTCAAGAAGGG-3′; reverse primer, 5′-CTCTGTACAACAT TCTCCTT-3′; β-actin forward primer, 5′-ATCATGTTTGAGACCTTCAACA-3′; reverse primer, 5′-CATCTCTTGCTCGAAGTCCA-3′.

### Western blot analysis

Equal amounts of protein (40–60 μg) were subjected to sodium dodecyl sulfate polyacrylamide gel electrophoresis (SDS-PAGE) and then transferred to polyvinylidene difluoride (PVDF) membranes. The membranes were blocked with 5% skim milk for 1 h at room temperature and then incubated at 4 °C overnight with the following primary antibody: anti-AKT1 (1:200; sc-81,434; Santa Cruz, CA, USA), anti-p-Aktser473 (1:500; sc-52,940; Santa Cruz), anti-c-myc (1:750; ab39688; Abcam Biotechnology, Cambridge, UK), anti-cyclin D1 (1:1000; sc-56,302; Santa Cruz); anti-β-actin (1:8000; ab3280; Abcam). The blots were then incubated with secondary anti-mouse (1:2000; cat. no. NA931V) and rabbit (1:2000; cat. no. NA934V) antibodies (Fisher Scientific, Pittsburgh, PA) for 2 h at room temperature. Protein bands were then detected using enhanced chemiluminescence (ECL) western blot detection reagents (Thermo Fisher Scientific) and analyzed by densitometry. Densitometric values, expressed as integrated optical intensity, were estimated in a CHEMIDOC XRS system by QuantiOne 1-D analysis software (Bio-Rad, Richmond, Calif., USA). The values obtained were normalized based on the densitometric values of internal β-actin and β-tubulin..

### Proliferation assay

Transfected cells were seeded in 96-well plates, at a density of 10^5^ cells/well in 200 μL of fresh media and incubated for 24, 48, and 72 h. At the end of incubation, 20 μL of 5 mg/mL 3-(4,5-dimethylthiazol-2-yl)-2,5-diphenyltetrazolium bromide assay (MTT, Sigma) were added to each well. The plates were incubated at 37 °C, under 5% CO_2_ for 4 h, following which 150 μL dimethyl sulfoxide (DMSO, Sigma) was added. The plates were gently agitated and the absorbance was measured at 490 nm wavelength using the Epoch Micro-plate Spectrophotometer (Bio-Rad, CA, USA).

### Colony formation assay

Transfected cells were seeded into 6 cm dishes at a density of 8 × 10^2^ cells cells/dish. After the next 2 weeks, cells were fixed with 4% PFA (Solarbio) for 15 min, then were stained with Giemsa (Solarbio) for 20 min, and washed twice with ddH_2_O. Visible colonies were manually counted under electron microscopy (Olympus, Japan).

### Cell cycle analysis

2 × 10^6^ cells/ml cells were seeded in a 6-well plate and were harvested 48 h after transfection. After 48 h, cells were collected and washed with Dulbecco’s phosphate buffered saline (DPBS; Genview, CA, USA), then fixed in 70% ethanol, and incubated overnight at 4 °C. The cell pellets were washed with DPBS followed by incubation with 300 μL of propidium iodide (PI; BD) solution for 30 min in the dark at 37 °C. Cells were then analyzed by flow cytometry (FCM, FACSCalibur; BD).

### Apoptosis assay

2 × 10^6^ cells/ml cells were seeded in 12-well plates and were harvested 72 h after transfection. According to the manufacturer’s instructions, the binding buffer, Annexin V/FITC and PI were added individually, followed by incubation in the dark at room temperature for 15 min. Apoptosis was then detected by FCM.

### Tumor xenograft model

All animal experiments in this study were performed according to the National Institutes of Health Guide for Care and Use of Laboratory Animals, and approved by the institutional ethical committee. Eight-week-old female BALB/c nude mice were purchased from the Shanghai Laboratory Animal Center of the Chinese Academy of Sciences (Shanghai, China) for use in in vivo studies. Cells (2 × 10^6^ cells/tumor) transfected with SNCG-RNAi vector (the SNCG siRNA group) or SNC-RNAi vector (the CON group) were injected subcutaneously into the right flank of nude mice. Tumor volume was measured every week, then mice were sacrificed, and tumors were harvested and weighed after 4 weeks.

### Statistical analysis

Each experiment was performed in triplicate. SPSS version 17.0 (SPSS Inc., IL, USA) was used for statistical analysis. All data were expressed as the mean ± SD, and the statistical differences among different groups were assessed by one-way analysis of variance. The two groups were compared using an independent samples t-test. Correlations of expression of SNCG and clinicopathological characters were analyzed by Pearson’s chi-square test. *P* < 0.05 indicated a significant difference, and *P* < 0.01 indicated that there was a very significant difference.

## Results

### *SNCG gene was up-regulated in cervical cancer.*

**Fig. 1:**
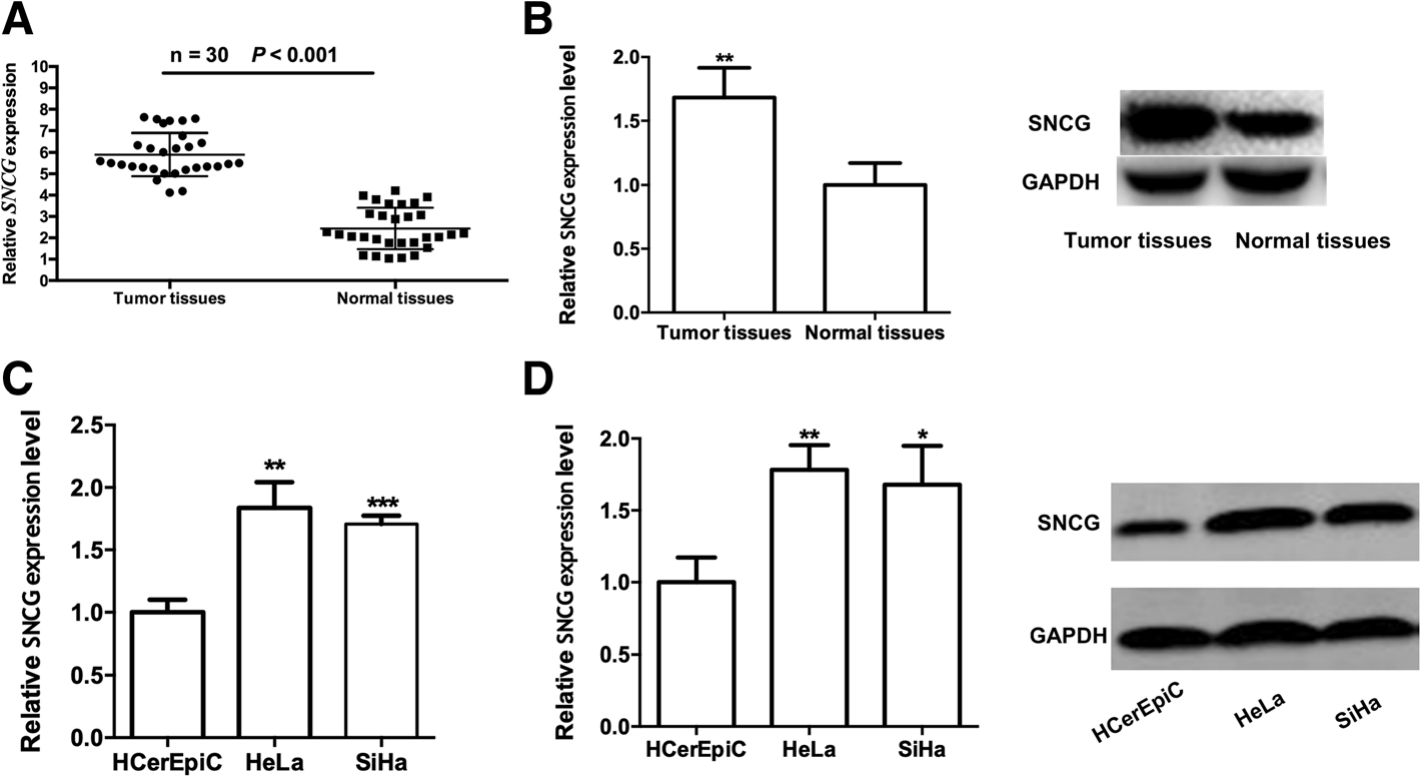
*SNCG gene was up-regulated in cervical cancer*. **a** Total RNAs were isolated from human cervical cancer tissues and adjacent non-tumor tissues. qRT-PCR was performed to determine the *SNCG* expression in human samples. **b** Protein expression level of SNCG in human cervical cancer tissues and adjacent non-tumor tissues. **c** qRT-PCR was performed to determine the SNCG expression in human cervical epithelial (HCerEpiC) cells in two human cervical cancer cell lines (HeLa, SiHa). **d** Protein expression levels of SNCG in human cervical cancer cell lines were detected. * *P* < 0.05, ** *P* < 0.01,*** *P* < 0.001

To investigate the role of *SNCG* in human cervical cancer, we explored the expression of *SNCG* in 30 cancer tissues and matched adjacent normal tissue. As Fig. 1a and b show, the mRNA and protein expression levels of *SNCG* were up-regulated in tumor tissues compared to normal tissues in *cervical cancer* (*P* < 0.01), which was in agreement with previous research, suggesting that *SNCG* might play a key role in *cervical cancer* development and progression. We then explored the expression level of SNCG in two human cervical cancer cell line (HeLa, SiHa) and one cervical epithelial cell (HCerEpiC) line. As Fig. 1c and d show, a higher SNCG expression level was observed in cancer cells than in the normal epithelial cells both at mRNA level and protein level (*P* < 0.05).

Since the SNCG increased with the progression of cervical cancer, we next estimated the correlation between the expression of SNCG and the clinicopathological characteristics of cervical patients. As shown in Table 1, the expression of SNCG had no association with age and histology. But patients with larger tumor size, FIGO stage, and lymph node metastasis (LNM) have higher-expressed SNCG (*P* < 0.05). These data implicated that, in patients with cervical cancer, SNCG could predict a poor clinical outcome including tumor size, FIGO, and LNM.

### *Silencing of SNCG inhibited proliferation of cervical cancer cells.*

**Fig. 2:**
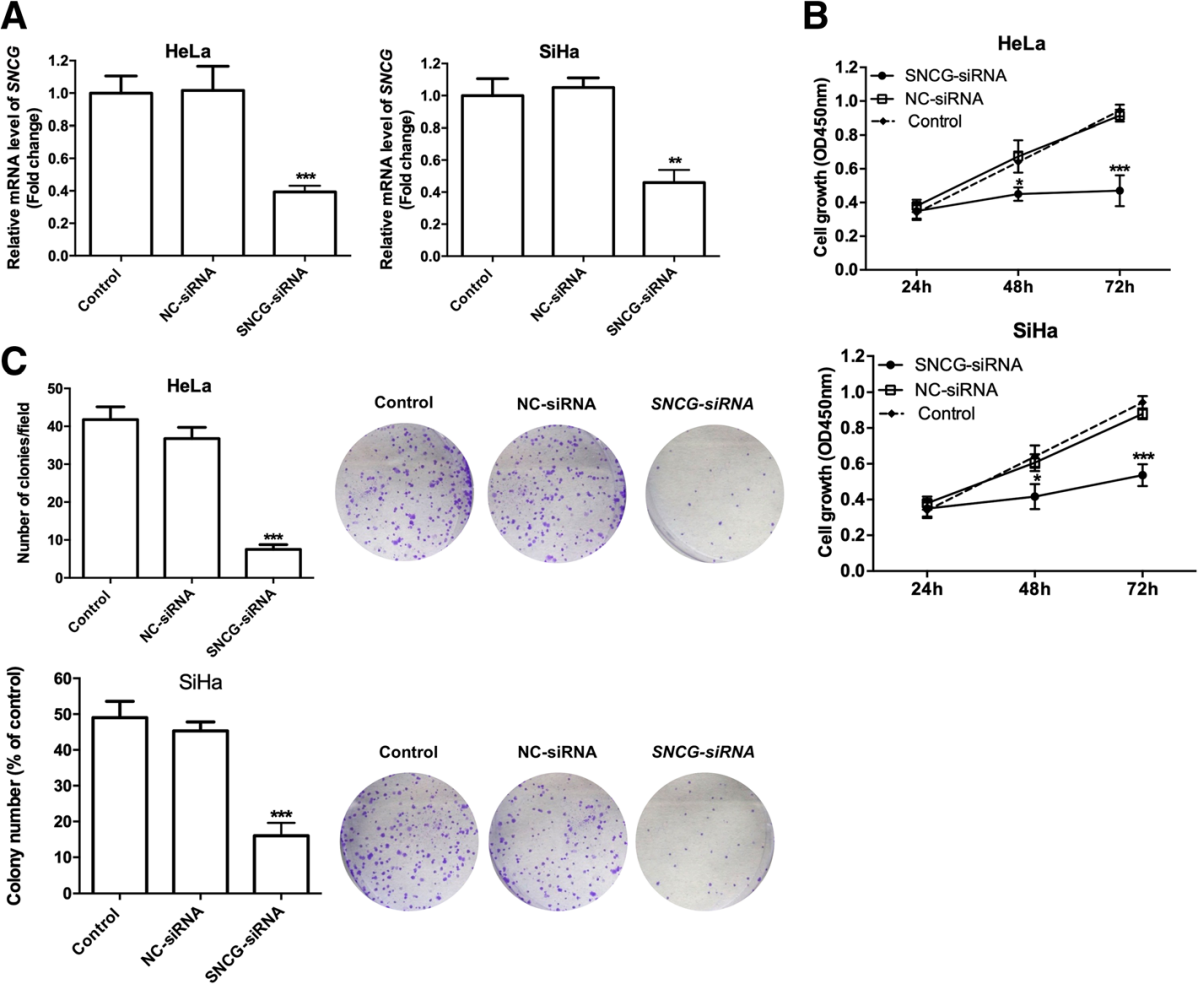
*Silencing of SNCG inhibited proliferation and transformation of cervical cancer cells* **a** SNCG-RNAi down-regulated SNCG mRNA expression in HeLa and SiHa cells. Total RNA was extracted and qRT-PCR was performed. **b** Effect of *SNCG* siRNA on cell growth determined by MTT assay. **c** Colony formation of HeLa and SiHa cells transfected with *SNCG* siRNA. * *P* < 0.05, ** *P* < 0.01,*** *P* < 0.001

To further understand the potential role of *SNCG in cervical cancer cells, we performed MTT assay and colony formation assay on cervical cancer cells with SNCG knockdown. As* Fig. 2 reveals, the *SNCG* expression was significantly inhibited 48 h after transfection with *SNCG* siRNA (*P* < 0.01, Fig. 2a). According to the growth curve of MTT assay, *SNCG siRNA* strongly decreased cervical cancer cell growth (*P* < 0.01, Fig. 2b). Furthermore, we confirmed the negative effect of *SNCG siRNA* by colony formation detection in which *SNCG siRNA group* cells displayed much smaller and fewer colonies than NC group cells and CON group cells (*P* < 0.01, Fig. 2c), indicating that *SNCG siRNA* had the ability to inhibit the growth and transformation ability of human cervical cancer.

### *Silencing of SNCG arrested* HeLa *cells in the G0/G1 phase and induced apoptosis.*

**Fig. 3:**
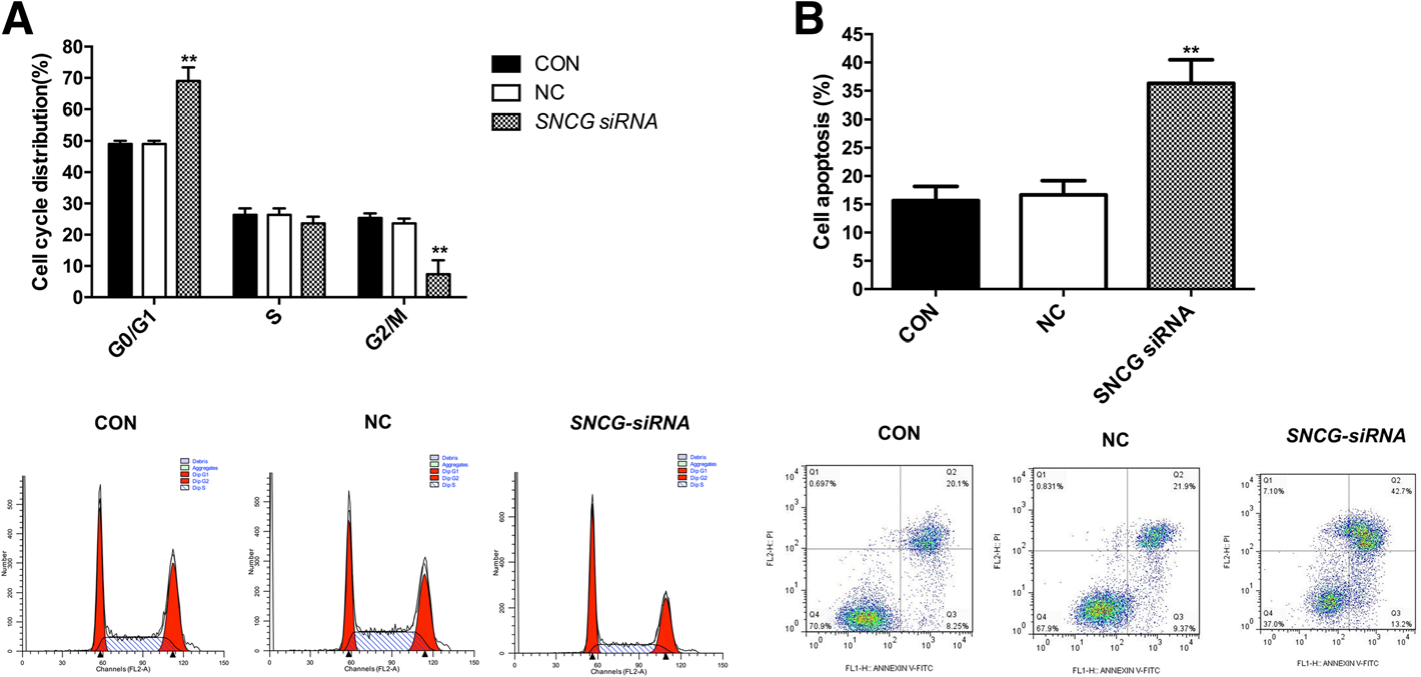
*Silencing of SNCG arrested HeLa cells in the G0/G1 phase* and induced apoptosis (**a**) The proportion of cells in G0/G1 phase significantly increased while the proportion in G2/M phase decreased in the *SNCG* siRNA group. **b** Detection of apoptosis 48 h after transfection with siSNCG using FCM analysis. SNCG RNAi significantly promoted cell apoptosis in the SNCG siRNA group. ** *P* < 0.01

Considering more obvious inhibitory effects on HeLa cells, we performed flow cytometry assay on HeLa cells with *SNCG* depletion. As shown in Fig. 3, *SNCG* depletion induced an obvious increase in the percentage of cells in G0/G1 phase in the *SNCG* siRNA group (Fig. 3a, *P* < 0.01), and an obvious decrease in the perscentage of cells in the G2/M phase (*P* < 0.01). Since *SNCG* depletion has been shown to induce apoptosis in certain cancer cells [7, 8], cell apoptosis analysis was performed, and the results indicated that the percentage of early and late apoptotic cell population increased to 13.2 and 42.7% respectively following *SNCG* depletion, much higher than that in the NC and CON groups (Fig. 3b, *P* < 0.01).

### *Silencing of SNCG inhibited growth of* cervical cancer *cells* in vivo

**Fig. 4:**
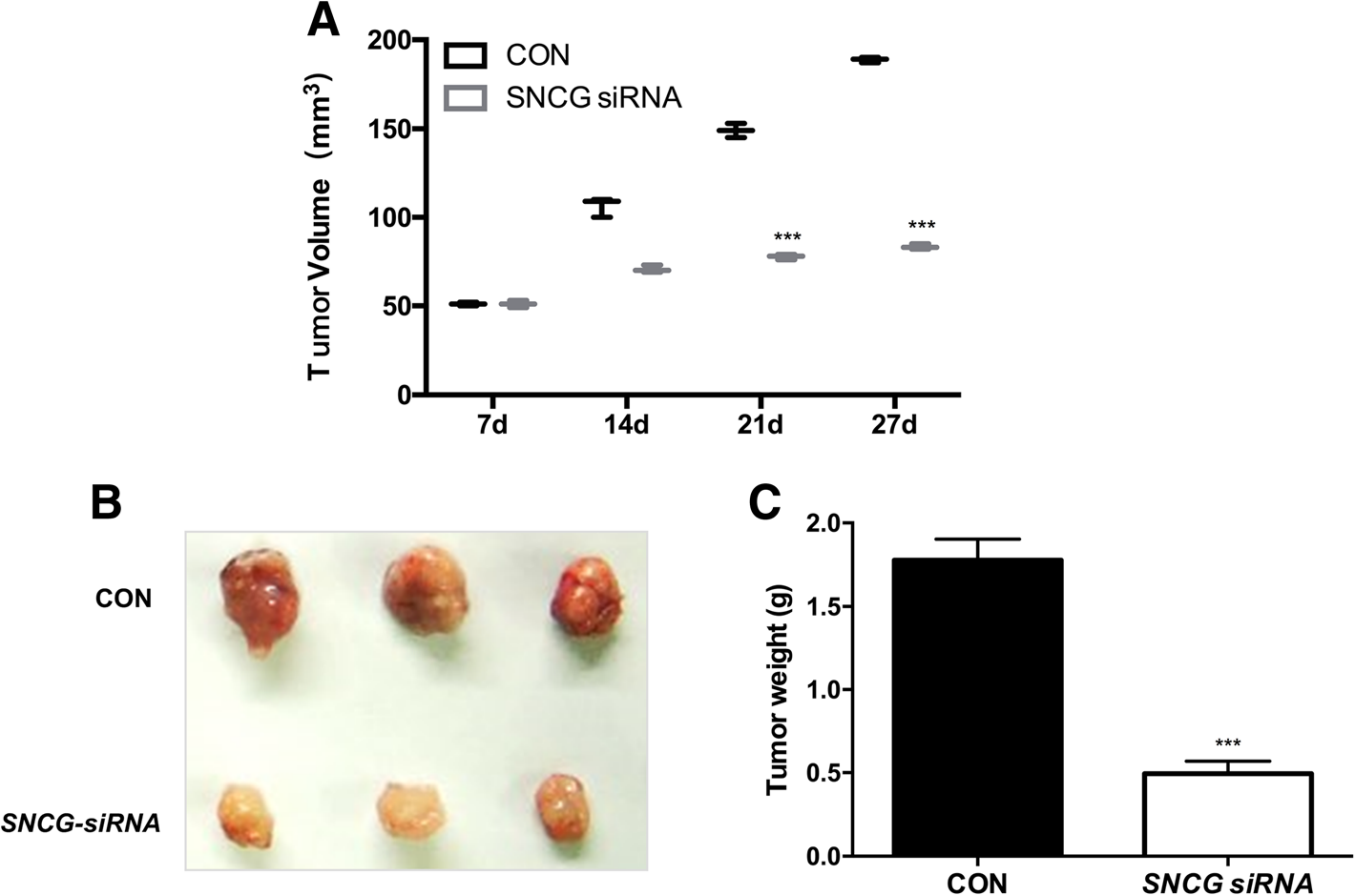
*Silencing* of *SNCG* inhibited the growth of cervical cancer cells in vivo **a** Tumors extracted at 27 days. **b** Weight curves of tumors at 27 days. Tumor weights were significantly decreased after *SNCG* siRNA. (***P < 0.001)

In a nude mouse tumor growth model, *SNCG* depletion significantly inhibited tumor growth, with decreased tumor weight and tumor size (Fig. 4, *P* < 0.001) in the *SNCG*-siRNA group compared with the CON group. Taken together, our research showed that *SNCG* might be an oncogene which promoted *cervical cancer tumorigenesis* in vitro *and* in vivo*.*

### Silencing of *SNCG* inhibited the *AKT* signaling pathways

**Fig. 5:**
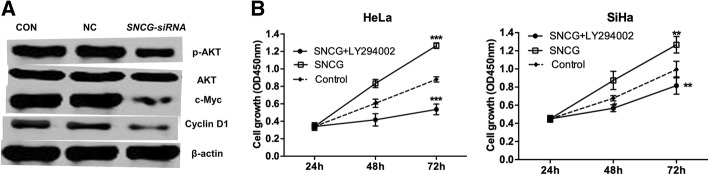
*Silencing of the SNCG gene affects the AKT signaling pathways.* **a** Western blotting of phosphorylated AKT (p-Akt), c-Myc, Cyclin D1 and their corresponding internal reference (β-actin). The levels of p-AKT, c-Myc, Cyclin D1 were lower in the SNCG siRNA group than NC and CON groups while there was no significant difference in expression of β-actin between the two groups. **b** Effect of *SNCG* on cell growth determined by MTT assay. LY294002 significantly reversed the upregulatory effect of SNCG on cell proliferation

Since *SNCG* knockdown contributed to *cervical cancer proliferation, we then explored the potential mechanism of SNCG in tumor development. The Akt signaling pathway plays an important role in cell proliferation regulation, and in our research, SNCG* siRNA significantly reduced Ser473 phosphorylated AKT (p-Akt) activation. Moreover, the expression levels of c-Myc and cyclin D1, which were the downstream targets of Akt signaling, were markedly reduced (Fig. 5a). In addition, treatment with the AKT inhibitor LY294002 (20 μM) for 6 h significantly blocked the effect of SNCG on cervical cancer growth (Fig. 5b), suggesting that AKT signaling was involved in SNCG-induced cervical cancer growth.

## Discussion

In 1997, Ji H et al. [9] demonstrated for the first time a breast cancer-specific gene (*BCSGC1*), which was expressed in high abundance in a breast cancer cDNA library but scarcely in a normal breast cDNA library, and it was identified as a putative breast cancer marker. *BCSG1* was also named *SNCG* or persyn due to sharing an identical gene sequence [8]*.* Since then, several studies have shown that *SNCG* was abnormally expressed in a high percentage of advanced and metastatic breast and ovarian tumors but not in normal or benign tissues [10, 11]. When over-expressed, *SNCG* has been primarily linked with increased cancer cell proliferation, chemoresistance and adverse outcomes in multiple solid tumors.

In the current study, we were interested in investigating the functional role of *SNCG* in cervical cancer. We transfected *SNCG* siRNA particles to silence *SNCG* gene expression, and cell proliferation decreased in cervical cancer cells infected with *SNCG* siRNA, indicating that *SNCG* indeed has the ability to promote cell growth. Previous studies showed that *SNCG* caused an increase in tumor growth in nude mice upon implantation of *SNCG*-upregulated cells [12, 13]. This was confirmed by our result that *SNCG* gene silencing caused decreased tumor growth in nude mice, indicating that *SNCG* has the ability to promote cervical cancer growth.

Various types of cancers have defects in the mitotic checkpoint, and previous studies have shown that ectopic expression of *SNCG* increased breast cancer cell growth through the mitotic checkpoint. This may provide a mechanism whereby over-expression of *SNCG* is an important driving force in tumor progression [14–16]. According to our results, the number of cells in the G0/G1 phase increased, while that in the S phase decreased, which suggested that down-regulation of *SNCG* was able to inhibit mitosis by blocking the cells in the G0/G1 phase. The G1 checkpoint plays an important role in cell damage repair since the cells with DNA damage will be blocked in the G1 phase; the damaged cells that cannot be repaired may directly undergo apoptosis [17], which is consistent with our results of the apoptosis analysis.

Previous studies have shown that *SNCG* promoted the expression of Akt and mTOR as induced cancer growth in human breast cancer [18]. SNCG siRNA played a significant role in the tumorigenesis of gastric cancer by downregulating the phosphorylation of AKT and ERK in human gastric cancer [19]. In our study, the change of AKT status in cells transfected with SNCG siRNA was investigated. SNCG depletion could decrease Ser473 phosphorylation of AKT, c-Myc and Cyclin D1, respectively. In addition, the PI3K/AKT inhibitor LY294002 could significantly reverse the upregulatory effect of SNCG on proliferation, which strongly supported the importance of SNCG in regulating cell proliferation via the AKT pathways.

## Conclusion

We found that down-regulation of *SNCG* expression inhibited cervical cancer cell growth in vitro and in vivo*,* which might be induced by the *Akt* signaling pathway. Therefore, *SNCG* is likely to play an important role in the progression of cervical cancer. Further studies are needed to determine whether *SNCG* will indeed make an effective biomarker for cervical cancer prognosis evaluation and therapy.

## Data Availability

Not applicable.

## Abbreviations

DPBS: Dulbecco’s phosphate buffered saline
ECL: Enhanced chemiluminescence
FCM: Flow cytometry
PI: Propidium iodide
PVDF: Polyvinylidene difluoride
SDS-PAGE: Sodium dodecyl sulfate polyacrylamide gel electrophoresis
siRNA: Small interfering RNA
